# High‐Performance Flexible Near‐Infrared Organic Light‐Emitting Diodes with a Heterogeneous Alloy Semitransparent Electrode

**DOI:** 10.1002/adma.202518212

**Published:** 2026-02-26

**Authors:** Mengxin Xu, Meina Han, Minying Xue, Shihao Liu, Yi Li, Gaoqiang Deng, Letian Zhang, Yuantao Zhang, Gang Cheng, Wenfa Xie, Chi‐Ming Che

**Affiliations:** ^1^ State Key Laboratory of Integrated Optoelectronics JLU Region College of Electronic Science and Engineering Jilin University Changchun China; ^2^ Department of Chemistry State Key Laboratory of Synthetic Chemistry HKU‐CAS Joint Laboratory on New Materials The University of Hong Kong Hong Kong SAR China; ^3^ Hong Kong Quantum AI Lab Limited Hong Kong SAR China

**Keywords:** external quantum efficiency, flexible NIR OLED, light outcoupling efficiency, magnesium‐bismuth alloy electrode, Purcell effect

## Abstract

Flexible near‐infrared (NIR) organic light‐emitting diodes (OLEDs) face efficiency challenges due to low photoluminescence quantum yields (PLQYs) in NIR emitters, governed by the energy gap law. Accelerating radiative transitions via the Purcell effect in optical microcavities offers a solution, but conventional flexible semitransparent electrodes struggle to balance microcavity‐enhanced PLQY and light outcoupling efficiency (OCE). We address this with a micro‐structured magnesium‐bismuth (Mg‐Bi) alloy electrode offering 40% broadband transmittance (400–1600 nm) and conductivity (29.3 Ω ◻^−1^). The alloy's low real permittivity supports less confined surface plasmon polariton and, with a capping layer, yields 60% NIR transmittance in an organic‐to‐air optical configuration. This design achieves 42.3% OCE and elevates the PLQY of a 704 nm NIR emitter to 71.8%, enabling flexible NIR‐OLEDs with a record 24.3% external quantum efficiency. The synergy of optical engineering and conductive microstructures establishes a universal strategy for high‐efficiency flexible NIR optoelectronics.

## Graphical abstract

This study presents a micro‐structured magnesium‐bismuth alloy electrode that enhances flexible NIR OLEDs. The electrode provides 40% broadband transmittance (400–1600 nm) and boosts light outcoupling efficiency (42.3%) and photoluminescence quantum yield (71.8%) through the Purcell effect in optical microcavities. This design achieves a record‐breaking 24.3% external quantum efficiency for flexible NIR OLEDs.

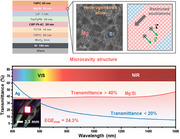

## 1 Introduction

Wearable medical electronics can revolutionize healthcare by enabling non‐invasive monitoring and treatment that is not constrained by time and location [1, 2, 3, 4]. For these devices, high‐performance near‐infrared (NIR) light sources are critical because they provide deep tissue penetration with minimal absorption and scattering—making them ideal for applications such as bio‐signal detection and phototherapy [5, 6, 7]. While conventional NIR light sources like incandescent or quartz halogen bulbs can serve as broadband emitters in stationary analytical and medical systems, they suffer from poor energy efficiency and limited spectral tunability. NIR light‐emitting diodes (LEDs), found in everything from remote controls to medical instruments [8], offer a mobile alternative; however, even when packaged for flexibility, they cannot match the inherent advantages of NIR organic LEDs (OLEDs) for wearable applications. OLEDs feature an ultrathin, mechanically compliant structure and are compatible with large‐area fabrication, allowing for seamless integration with the human body [9, 10].

Currently, despite progress in optimizing conjugated systems and substituents, the photoluminescence quantum yield (PLQY) of NIR emitters is significantly lower than that of visible emitters, limiting the potential of OLEDs as efficient NIR light sources [11, 12, 13]. The fast non‐radiative decay pathway governed by the energy gap law mainly causes this limitation [14]. While mitigating nonradiative processes is crucial, enhancing the radiative transition rate is a promising strategy to increase the PLQY of NIR emitters and thus improve the external quantum efficiency (EQE) of NIR OLEDs. Integrating a microcavity structure with a semi‐transparent electrode is a proven strategy to enhance the radiative transition rate via the Purcell effect [15]. For such structures in the visible range, silver (Ag) is the material of choice for the flexible semi‐transparent electrode [15, 16]. Nevertheless, its applicability to NIR devices is severely limited. While a continuous Ag film with a thickness >15 nm—optimized for visible microcavities—can achieve a transmittance of ∼60% in the visible region, its transmittance drops to less than 15% in the NIR range [17]. This is because the frequency of NIR light is low, making it easier for free electrons in the metal to follow the electric field oscillations, thereby enhancing the metal reflection in this range [18]. The enhanced reflection not only causes the light to undergo additional reflections and absorptions before extraction, which adversely affects the outcoupling efficiency (OCE), but also introduces a significant angular dependence in the NIR emission. Therefore, developing a novel semitransparent metal electrode that balances PLQY enhancement with high OCE is crucial for advancing microcavity NIR OLEDs.

Here, we introduce a heterogeneous alloy approach to create semitransparent magnesium (Mg)‐based electrodes for flexible, high‐performance NIR OLEDs. By adding low‐conductivity bismuth (Bi) to conductive Mg, it forms a mesh‐like Mg structure around the Bi core, which dampens charge oscillations, reduces NIR reflection, and enhances transmittance. The Mg‐Bi (MB) alloy electrode shows excellent conductivity with a sheet resistance of 29.3 Ω ◻^−1^, significant bending resistance, strong adhesion to polymer substrate, and a transmittance of ∼40% over a wide wavelength range of 400–1600 nm. When integrated into a flexible OLED with an additional capping layer (CL), the transmittance further improves to around 60% in the organic‐to‐air optical configuration. This enhancement increases the PLQY of the NIR emitter to 71.8% and the OCE to 42.3%, resulting in a record EQE of 24.3% at 704 nm. This performance exceeds that of most state‐of‐the‐art NIR OLEDs on glass substrates [19, 20, 21, 22, 23, 24, 25, 26, 27, 28, 29, 30, 31].

## 2 Result and Discussion

### 2.1 Flexible OLEDs with Mg:Bi Alloy Semitransparent Electrode

**FIGURE 1:**
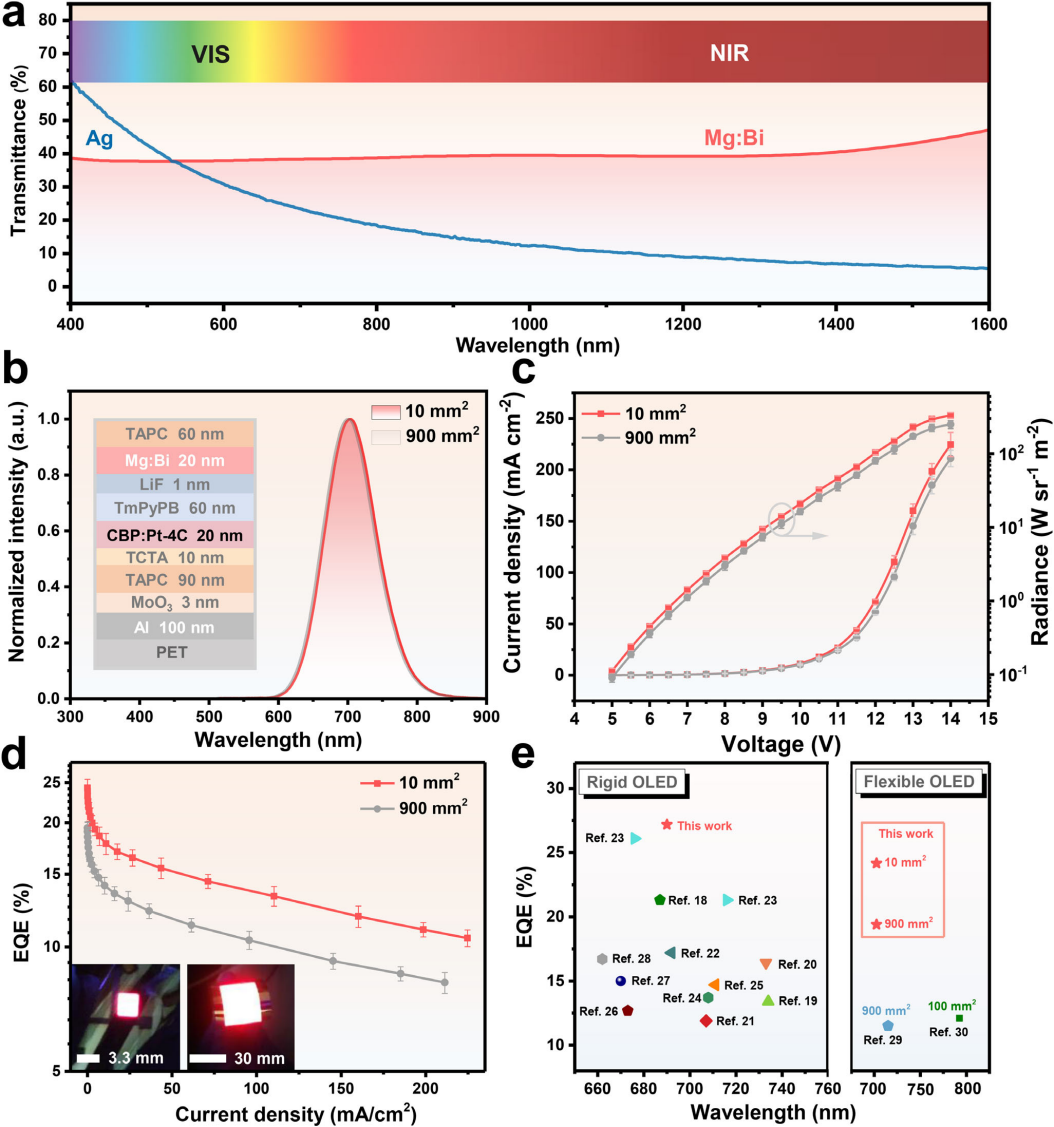
Performance of flexible OLEDs with Mg:Bi alloy semitransparent electrodes. (a) Optical transmittance of 20 nm films of pure Ag and Mg:Bi alloy, measured across the wavelength range of 400–1600 nm. (b) Device structure and EL spectra, (c) J‐V‐R characteristics, and (d) EQE‐J characteristics for flexible OLEDs with emitting areas of 10 and 900 mm^2^. The inset shows photographs of the devices during operation. (e) Comparison of EQE values for Mg:Bi‐based OLEDs with previously reported rigid and flexible NIR OLEDs.

Ag is commonly used as an electrode in OLEDs due to its high conductivity and suitable work function. Ultrathin and discontinuous Ag films can achieve high transmittance in the visible and NIR ranges [32]. However, as shown in Figure 1a, the 20 nm thick continuous Ag film exhibits relatively low transmittance in the NIR region. To overcome this limitation, we developed a Mg‐based heterogeneous alloy (Mg:Bi alloy) by incorporating 20 wt.% of low‐conductivity Bi into Mg. This 20 nm Mg:Bi alloy achieves a transmittance of approximately 40% over a wide wavelength range from the ultraviolet to NIR, effectively doubling the NIR transmittance of a 20 nm Ag film (20%), as shown in Figure 1a. Using this Mg:Bi alloy as a semi‐transparent electrode, we successfully fabricated NIR OLEDs with emission areas of 10 mm^2^ and 900 mm^2^ on flexible PET substrates (Figure 1b–d). Figure 1b provides details of the device structure and emission spectrum, while Figure 1c,d show the current density‐voltage‐radiance (J‐V‐R) and EQE–current density (EQE‐J) characteristics, respectively. The inset of Figure 1d shows images of the two devices during operation. The 10 mm^2^ device achieves a maximum radiance of 332.6 W sr^−1^ m^−2^ and a maximum EQE of 24.3% at 704 nm, while the 900 mm^2^ device achieves a maximum radiance of 250.1 W sr^−1^ m^−2^ with a still high maximum EQE of 19.7%. To the best of our knowledge, this represents the state‐of‐the‐art performance for flexible NIR OLEDs and is even comparable to the best‐performing rigid NIR OLEDs, as shown in Figure 1e [19, 20, 21, 22, 23, 24, 25, 26, 27, 28, 29, 30, 31].

### 2.2 Mechanism of NIR Transmittance Enhancement in Mg:Bi Alloys

**FIGURE 2:**
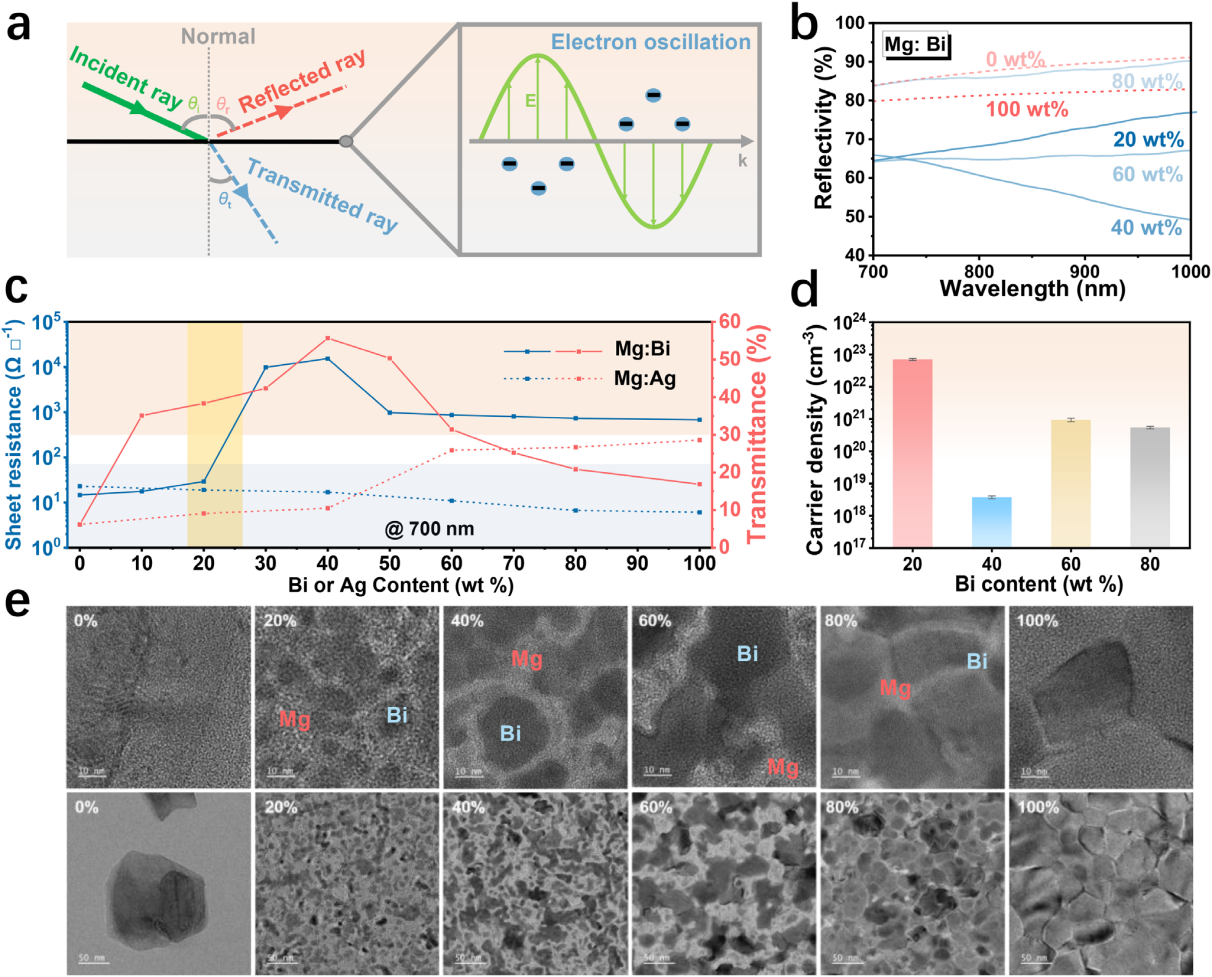
Mechanism of NIR transmittance enhancement in Mg:Bi alloy films. (a) Schematic diagram illustrating light transmission and reflection. The enlarged section highlights electron oscillations induced by the interaction between light and metal. (b) NIR reflectivity of Mg:Bi alloys between 700 and 1000 nm as a function of Bi ratio, measured at a 55‐degree incident angle. (c) Dependence of NIR transmittance at 700 nm and sheet resistance on Bi or Ag ratio in Mg‐based alloys. (d) Charge carrier density in Mg:Bi alloys with Bi ratios of 20, 40, 60, and 80 wt.%, determined via Hall effect measurements. (e) TEM images of the Mg:Bi alloy at two magnifications (top: scale bar = 10 nm, bottom: scale bar = 50 nm).

To understand the alloy mechanism behind the transmittance enhancement, we fabricated Mg‐based alloys by doping with high‐conductivity Ag (Mg:Ag alloy) and low‐conductivity Bi (Mg:Bi alloy). The thickness of the two alloy films, as well as that of subsequent films, is 20 nm and will not be specified further unless otherwise noted. As shown in Figure S1a,b, the two alloys show distinct transmittance changes with the change of doping ratio. The transmittance of the Mg:Ag alloy lies between that of pure Mg and pure Ag, whereas the transmittance of the Mg:Bi alloy is significantly higher than that of both pure Mg and pure Bi. In optics, the sum of transmitted, reflected, and absorbed light equals the total incident light, as shown in Figure 2a. Previous studies have shown that Mg films have high reflectivity and low absorptivity in the NIR range [33]. Therefore, the enhanced NIR transmittance observed in Mg:Bi alloys may be due to the reduced reflection compared to pure Mg and pure Bi films. Figure 2b shows the reflectivity of the Mg:Bi alloy measured at an incident light angle of 55°. As the Bi ratio increases from 0 to 100 wt.%, the reflectivity of the Mg alloy in the NIR range first decreases and then increases, while the transmittance first increases and then decreases (Figure S1b). The 40 wt.% Bi alloy has the lowest reflectivity and the highest transmittance. Therefore, the observed increase in the NIR transmittance of the Mg alloy can be attributed to the suppression of reflection.

When light waves strike a metal, they induce oscillations in free electrons, producing secondary waves with a phase shift of 180°, resulting in specular reflection. This mechanism implies that the suppression of optical reflection is closely linked to the behavior of free charge carriers. To explore this relationship, we investigated the NIR transmittance and electrical conductivity of Mg‐based alloys with varying compositions (Figure 2c). In Mg:Bi alloys, as the Bi content increases, the sheet resistance follows a trend similar to that of the NIR transmittance. At 40 wt.% Bi, the sheet resistance reaches a maximum, while the transmittance also peaks at 55% at 700 nm. In contrast, Mg:Ag alloys exhibit conductivity values that fall between those of pure Mg and Ag—consistent with their NIR transmittance behavior—thereby underscoring the correlation between electrical conductivity and optical transmission in these alloy films as well.

To further understand the role of charge carriers, we conducted Hall measurements to determine both the carrier density and mobility in Mg:Bi alloys (Figure 2d and Figure S2). As the Bi content increases from 20 to 40 wt.%, the carrier concentration drops sharply from ∼7 × 10^22^ cm^−3^ to ∼4 × 10^18^ cm^−3^. With further increases to 60 and 80 wt.% Bi, the carrier concentration rises again to ∼9 × 10^20^ cm^−3^ and ∼6 × 10^20^ cm^−3^, respectively. Meanwhile, the Hall mobility shows an inverse trend relative to carrier density. The observation that the 40 wt.% Bi alloy has both the highest sheet resistance (and thus lowest conductivity) and the highest carrier mobility is identified as a signature of a disorder‐driven metal‐insulator transition (MIT). This Bi‐concentration‐dependent MIT is further supported by the temperature‐dependent resistance behavior: the resistance of the 40 wt.% Bi alloy film decreases with increasing temperature, indicative of semiconducting behavior, in contrast to metallic films, for which the resistance typically increases with temperature (Figure S3). Across such a transition, alloy disorder converts extended states into localized ones, leading to a sharp, nonlinear collapse in carrier concentration near the transition [34, 35]. The electronic states that localize first are those with unfavorable energies, larger effective masses, and intrinsically low mobility. The states that remain extended possess more favorable energies, are more delocalized, and experience weaker scattering. Consequently, the average mobility of the remaining carriers can remain unchanged or even increase [36]. These results indicate that the enhancement in NIR transmittance is primarily governed by the reduction in carrier density rather than by limitations in carrier mobility.

To further investigate the structural disorder underlying the carrier‐density variations, we acquired high‐resolution TEM images of the Mg:Bi alloys (Figure 2e). In the TEM image, Mg appears as a bright area due to its low atomic number and weak electron scattering, while Bi appears as a darker area due to its high atomic number and strong electron scattering. The pronounced contrast observed in TEM images of Mg:Bi alloys with Bi ratios of 20, 40, and 60 wt.% indicates that Mg and Bi form a heterogeneous, rather than a homogeneous, alloy. This heterogeneous microstructure is further supported by X‐ray diffraction analysis (Figure S4). The diffraction pattern of the 20 wt.% Bi alloy exhibits distinct Mg peaks superimposed on a broad amorphous background, consistent with a heterogeneous structure in which Bi incorporation disrupts the long‐range order of the Mg lattice. In contrast, the 80 wt.% Bi alloy shows sharp, phase‐pure Bi diffraction peaks, confirming the absence of the desired heterogeneous alloy structure. In addition, selected‐area electron diffraction patterns of the Mg:Bi alloys (Figure S5) can be indexed exclusively to polycrystalline Mg or Bi, thereby excluding the formation of other intermetallic phases during the co‐evaporation process.

**FIGURE 3:**
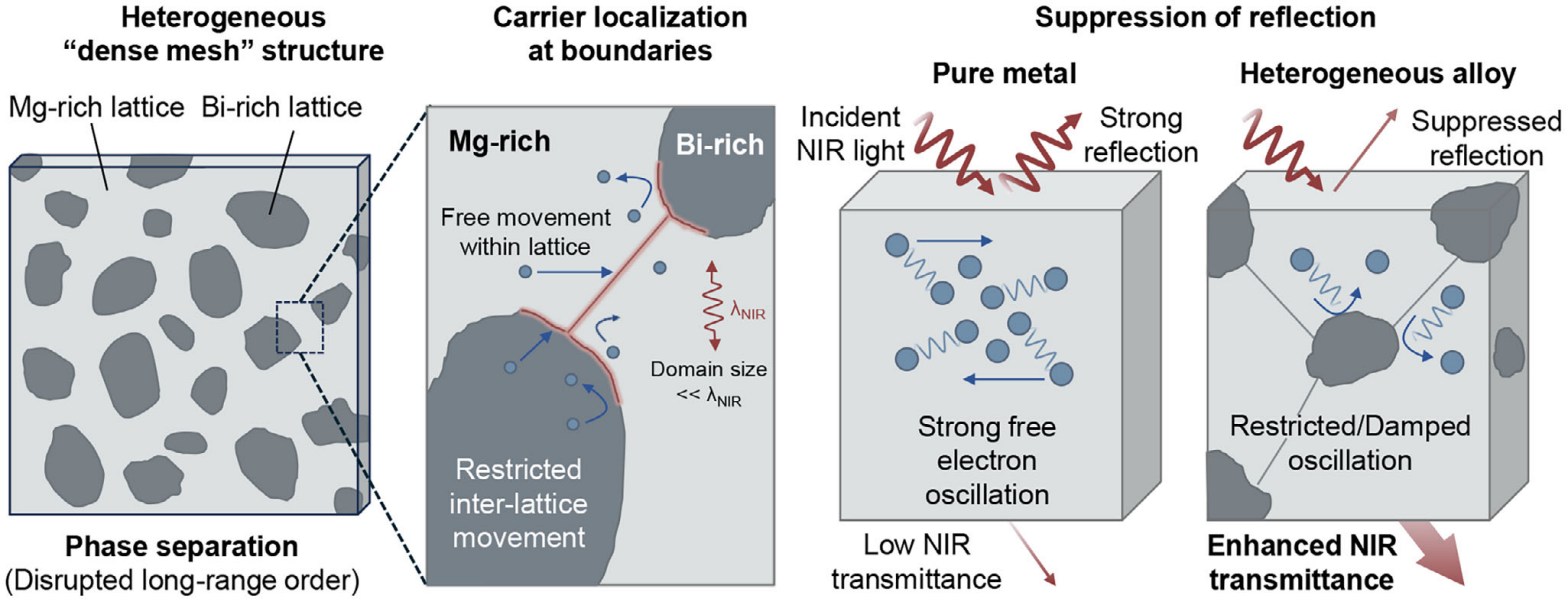
Mechanism of enhanced NIR transmittance through restricted carrier oscillations in heterogeneous alloy distributions.

Based on the above observations, a mechanism is proposed to explain the carrier density variations and the enhancement of NIR transmittance (Figure 3). In the heterogeneous structure, a dense mesh is formed, with Bi atoms clustered at the core and Mg atoms surrounding them. It is believed that carriers can move freely within the Mg or Bi lattices because pure Mg and Bi films exhibit low sheet resistances of 14.7 and 679.1 Ω ◻^−1^, respectively. In contrast, the sheet resistance of the alloy film reaches a peak value of 15.5 kΩ ◻^−1^ when the alloy composition is close to the equilibrium ratio, so the carrier movement between Mg and Bi lattices is severely restricted. Based on this, when light waves are incident on the metal, the oscillations of free electrons in the Mg or Bi lattice are restricted in certain directions because the size of the Mg or Bi lattices is relatively small compared to the NIR wavelength. This limitation results in reduced NIR reflection and enhanced transmittance. On the other hand, when the alloy composition approaches an equilibrium ratio, the connection between Mg lattices is hindered by Bi lattices, and vice versa. Therefore, a suitable alloy ratio is required to maintain a connected network of Mg or Bi lattices to enable the movement of free electrons. Due to the high electrical conductivity of Mg, the sheet resistance remains below 30 Ω ◻^−1^ when the Bi ratio is below 20 wt.%. When the Bi ratio is 20 wt.%, an ideal NIR semi‐transparent electrode can be achieved, balancing the transmittance (38.3% at 700 nm) and conductivity (29.3 Ω ◻^−1^).

### 2.3 Performance Improvement in NIR OLEDs with Mg:Bi Alloy

**FIGURE 4:**
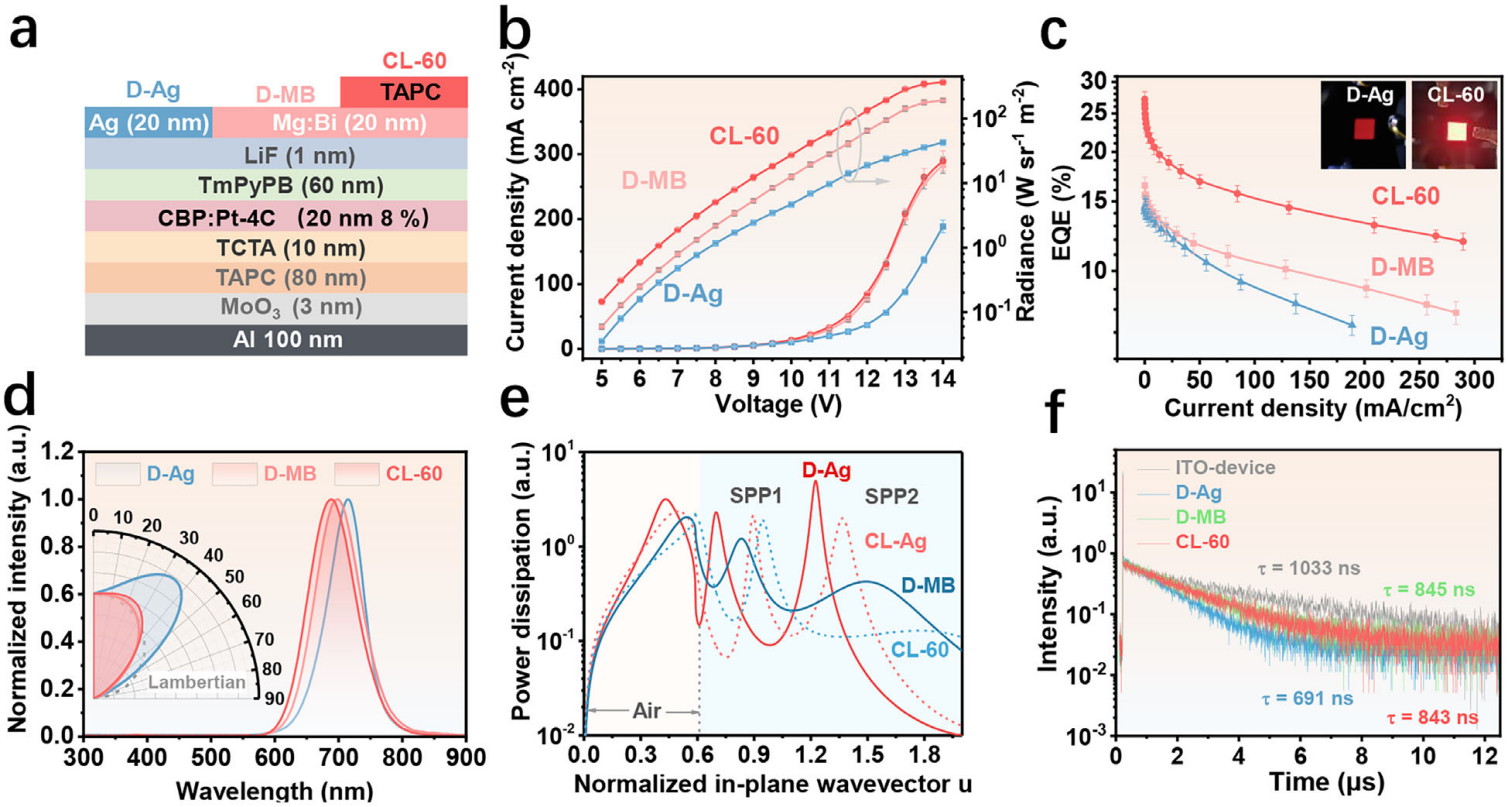
EL performance of rigid NIR OLEDs with Ag and Mg:Bi alloy cathode. (a) Schematic representation of the device structure, (b) J‐V‐R characteristics, (c) EQE‐J characteristics, (d) EL spectra and angular emission profiles of D‐Ag, D‐MB, and CL‐60 devices. (e) Power dissipation characteristics of devices D‐Ag and D‐MB, along with their corresponding capping‐layer‐modified counterparts, CL‐Ag and CL‐60. (f) transient PL decay characteristics of ITO‐based, D‐Ag, D‐MB, and CL‐60 devices.

To validate the proposed Mg:Bi alloy film as an effective semitransparent electrode for NIR OLEDs, we fabricated a NIR OLED with it as the top cathode, referred to as device D‐MB. For comparison, a device with a conventional semitransparent Ag cathode was also prepared, labeled as device D‐Ag. Both devices have the same structure (Figure 4a) but different cathodes, and both utilize a highly efficient Pt‐based NIR emitter, Pt‐4C [19]. The device structure consists of Al (100 nm)/MoO_3_ (3 nm)/Di‐[4‐(N,N‐di‐p‐tolyl‐amino)‐phenyl]cyclohexane (TAPC, 80 nm)/4,4′,4′′‐tri(N‐carbazolyl)‐triphenylamine (TCTA, 10 nm)/ 4,4'‐bis(carbazol‐9‐yl)biphenyl (CBP): 8 wt.% Pt‐4C (20 nm)/1,3,5‐tri[(3‐pyridyl)‐phen‐3‐yl] benzene (TmPyPB, 60 nm)/LiF (1 nm)/cathode (20 nm). Figure 4b,c shows the J‐V‐R and EQE‐J characteristics of these devices. As shown in Figure 4b, under the same bias, D‐MB exhibits a higher current density than D‐Ag. This is because the work function of Mg is low, which is conducive to enhancing electron injection [37, 38]. Thus, a broader operating current density window is achieved for D‐MB compared to D‐Ag. Specifically, the current density at which maximum radiance occurs increases from 188.7 mA cm^−2^ in D‐Ag to 283.2 mA cm^−2^ in D‐MB. Furthermore, D‐MB achieves a maximum EQE of 16.5%, exceeding the 14.6% of D‐Ag. This combination leads to an increase in maximum radiance from 42.6 W sr^−1^ m^−2^ (D‐Ag) to 190.9 W sr^−1^ m^−2^ (D‐MB), highlighting the advantage of enhanced electron injection in maintaining high‐efficiency operation at elevated current densities.

As shown in Figure 4d, D‐Ag exhibits noticeable spectral narrowing, shift, and super‐Lambertian distribution, while D‐MB shows reduced spectral narrowing and near Lambertian emission. This indicates that the light emitted by the NIR emitter inside the D‐Ag undergoes multiple reflections between the Al and Ag mirrors that form the optical cavity. In addition to the super‐Lambertian distribution, the high reflectivity of the Ag cathode also leads to a significant wavelength shift with changing viewing angle, as shown in Figure S6a. When the viewing angle shifts from 0° to 80°, D‐Ag exhibits a blue shift of 65 nm. In contrast, the reduced reflectivity of the Mg:Bi alloy cathode in D‐MB results in a much smaller blue shift of 32 nm in the same angular range (Figure S6b). To further reduce the wavelength shift and enhance light OCE, a 60 nm TAPC CL was introduced atop the cathode in both D‐Ag and D‐MB, yielding devices labeled CL‐Ag and CL‐60 (Figure S7 and Figure 4a), respectively. As shown in Figure 4b, the effect of adding CL on the current density is negligible. However, it enhances electrode transmittance through optimized optical interference at the dielectric‐metal interface (Figure S8). With the CL, the radiance of CL‐60 is significantly enhanced, reaching a peak radiance of 365.4 W sr^−1^ m^−2^ which is about 1.6 times that of D‐MB and notably greater than that of D‐Ag (inset of Figure 4c). As a result, CL‐60 achieved a peak EQE of 27.1% at 690 nm, surpassing all previously reported values in this wavelength range (Figure 1e) [19, 20, 21, 22, 23, 24, 25, 26, 27, 28, 29]. Additionally, the peak wavelength of CL‐60 shifted only 15 nm when the angle changed from 0° to 80° (Figure S9), showing an improvement in angular stability. As further illustrated in Figure S10, CL‐60 achieves a 40% higher peak EQE than CL‐Ag (27.1% vs 19.2%). This improvement arises from the Mg:Bi alloy's optical properties: unlike Ag, which strongly confines SPPs due to its large negative permittivity (Figure S11), the Mg:Bi alloy—with a much lower real permittivity—supports less confined SPP modes that can be more efficiently outcoupled when a capping layer is introduced.

To understand the underlying mechanism, we simulated the power dissipation spectra of D‐Ag, D‐MB, CL‐Ag, and CL‐60 using our home‐made software OptiXLED based on the classical electromagnetic theory [15], as shown in Figure 4e. Compared with D‐Ag, D‐MB exhibits suppressed SPP1 and SPP2 modes, corresponding to the hybrid surface plasmon polariton (SPP) modes of the bottom and top electrode, respectively. This resulted in an increase in OCE from 20.0% for D‐Ag to 25.4% for D‐MB. Compared with D‐MB, the addition of the CL effectively suppressed the SPP2 mode. This indicates that CL helps to suppress the SPP2 mode and enhance radiative coupling into the air mode at the top electrode, thereby improving the OCE of CL‐60 to 42.3%. In contrast, while the CL also suppresses SPP2 in CL‐Ag, residual mode strength persists due to Ag's inherent plasmonic properties, limiting OCE enhancement to 26.4%.

In addition to OCE, we also studied the effect of the optical microcavity of Mg‐Bi alloy and Ag electrode on the exciton lifetime of Pt‐4C emitter (Figure 4f). Compared with the long exciton lifetime of 830 ns in the ITO‐based device, the exciton lifetimes in the devices containing D‐Ag, D‐MB, and CL‐60 are shortened to 410, 607, and 633 ns, respectively. With the acceleration of the radiative transition, the PLQY of the Pt‐4C emitter in D‐Ag, D‐MB, and CL‐60 is inferred to increase from its intrinsic value of 63% to 81.7%, 72.9%, and 71.8%, respectively. However, experiments without the reflective Al electrode revealed a critical difference (Figure S12): the D‐MB device's lifetime reverted to nearly that of the ITO device, while the D‐Ag device's lifetime remained short. This indicates that the rapid decay in D‐Ag is dominated by non‐radiative SPP coupling to the Ag electrode, whereas in D‐MB, it is caused by the radiative Purcell effect. Therefore, the reported PLQY enhancement in D‐Ag is overvalued, unlike the genuine enhancement in D‐MB. Overall, analysis using the equation EQE = γ × PLQY × OCE confirms that the higher EQE in Mg‐Bi‐based OLEDs results from concurrent improvements in both OCE and PLQY.

To assess the generalizability of Mg:Bi electrodes for NIR OLEDs, we fabricated devices using a thermally activated delayed fluorescence emitter APDC‐DTPA (3,4‐bis(4‐(diphenylamino)phenyl)acenaphtho[1,2‐b]pyrazine‐8,9‐dicarbonitrile) and a phosphorescent emitter Pt 2–46, with emission peaks beyond 750 and 850 nm, respectively. These devices were compared with Ag‐based counterparts (Figures S13 and S14). The Mg:Bi‐based devices showed superior radiance and EQE. Notably, the APDC‐DTPA device with Mg:Bi achieved a peak EQE of 3.5% at 751 nm—1.49 times higher than the Ag‐based devices and 1.63 times higher than prior literature values for the same emitter [39]. Similarly, the Mg:Bi device with Pt 2–46 demonstrated enhanced performance, achieving a peak EQE of 2.8% at 860 nm compared to 1.9% for the corresponding Ag‐based device.

### 2.4 Flexible NIR OLEDs with Mg:Bi Electrodes for Wearable Applications

**FIGURE 5:**
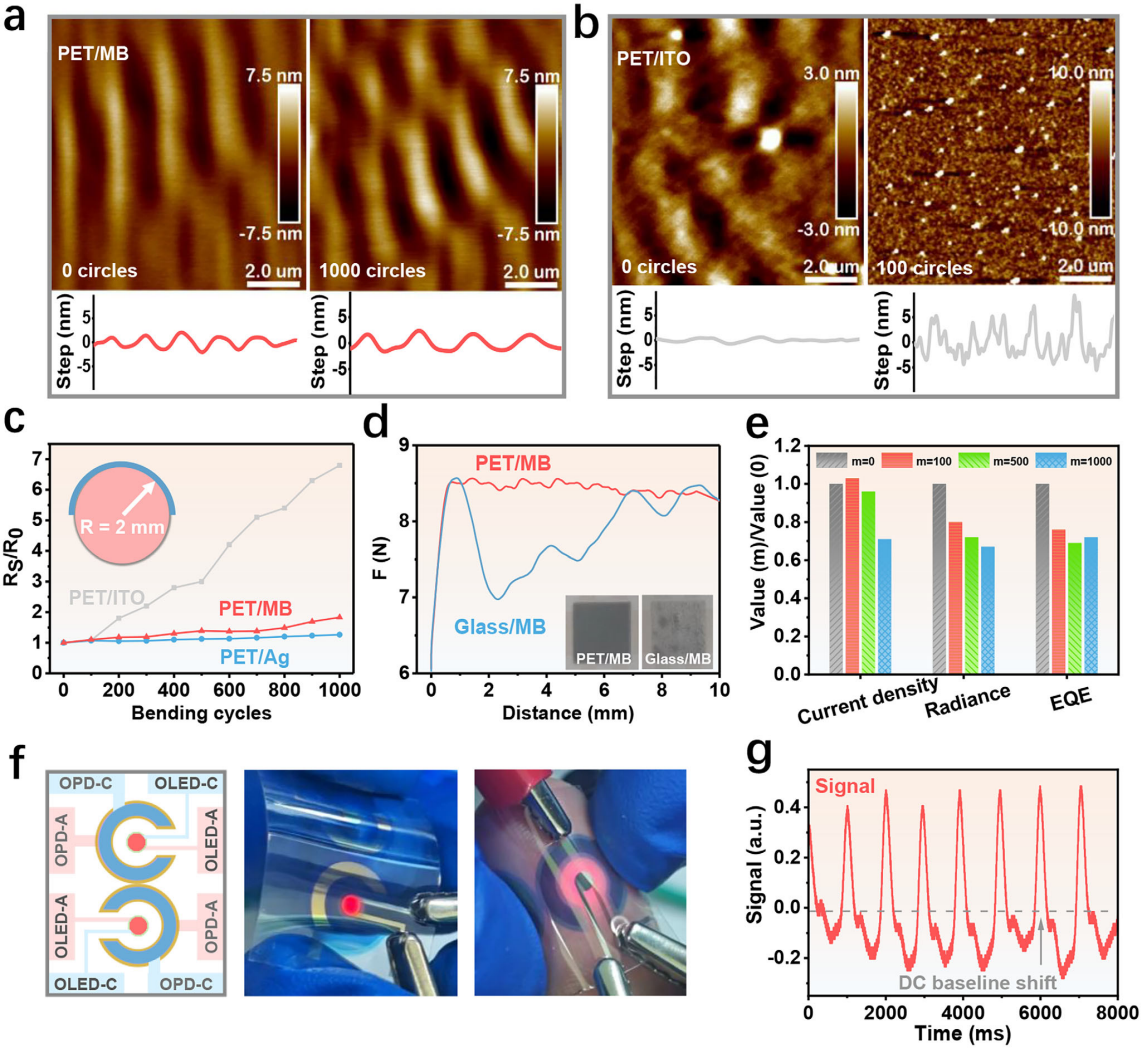
Flexibility of flexible NIR OLEDs with Mg:Bi alloy semitransparent electrode. AFM surface morphology (top) and height profiles (bottom) of (a) PET/MB and (b) PET/ITO films before and after bending. (c) Changes in sheet resistance of PET/Ag, PET/MB, and PET/ITO films after bending. (d) Adhesion strength of Mg:Bi films on PET and glass substrates, with inset images showing the films after peeling tests. (e) Variations in current density, radiance, and EQE before and after bending. All bending cycles were performed with a 2 mm radius. (f) Proposed PPG sensor structure (left) photograph during operation (middle and right). (g) Pulse signals captured by the designed PPG sensor.

While the previous section, Flexible OLEDs with Mg:Bi Alloy Semitransparent Electrode, detailed the optoelectronic properties of Mg:Bi‐based flexible OLEDs, we evaluate here their mechanical robustness. Using PET film as a flexible substrate, 20 nm Ag and Mg:Bi films were deposited, denoted as PET/Ag and PET/MB, respectively. Figure 5a,b are the atomic force microscopy (AFM) images of PET/MB and PET/ITO after multiple bending cycles, respectively. Although PET itself wrinkles upon bending (Figure S15), the PET/MB electrode maintains a continuous and intact surface morphology even after 1000 bending cycles, while the morphology of ITO is severely degraded after only 100 bends. Figure 5c illustrates the sheet resistance changes of these two electrodes and a commercial PET/ITO electrode as a function of bending cycles (radius = 2 mm). The sheet resistance of the PET/ITO electrode after 1000 bending cycles reached almost seven times the initial value, indicating the fragility of ITO. In contrast, the sheet resistance of PET/Ag and PET/MB increased only slightly after 1000 bending cycles, indicating that they have strong bending resistance. These results highlight the potential of Mg:Bi alloys as semitransparent electrodes for flexible optoelectronic applications.

In addition to being resistant to bending, the Mg:Bi alloy films also exhibit strong adhesion to polymer substrates, highlighting their potential for long‐term mechanical stability in flexible optoelectronics. To evaluate the adhesion, we attached the same area of tape to the metal surfaces of PET/MB and Glass/MB, and measured the pull force required to peel off the tape over a specified displacement, as shown in Figure 5d. As the displacement increases, the peeling force between the Mg:Bi film and the glass decreases significantly, indicating that Mg:Bi is detached from the glass. In contrast, the Mg:Bi electrode exhibited strong adhesion to PET, with the peeling force remaining nearly constant regardless of the displacement. These results are consistent with the electrode photographs after the peeling test shown in the inset image. Subsequently, the durability performance of the 10 mm^2^ flexible NIR OLED after bending was analyzed. As shown in Figure 5e, even after 1000 bending cycles with a 2 mm radius, the device can still maintain more than 70% of its initial current density, radiance, and EQE, demonstrating its excellent flexibility. Performance can be further improved by using thinner, more flexible substrates with lower bending stiffness.

Finally, the integration of Mg:Bi‐based NIR OLEDs and OPDs (device structure and performance details in Figure S16) into a flexible photoplethysmography (PPG) sensor was explored, as shown in Figure 5f. NIR light offers excellent tissue penetration and experiences less attenuation in the skin and subcutaneous adipose tissue. Its transmission intensity is approximately five times higher than that of green light, enabling a higher signal‐to‐noise ratio and the ability to detect signals from deeper tissue layers [40, 41]. As shown in Figure 5g, the PPG sensor effectively captures physiological signals from pulsating blood vessels, presenting a clear and regular pulse waveform. The minimal DC drift observed (signal centered near zero baseline) highlights the efficacy of Mg:Bi‐based NIR OLEDs, whose deep tissue penetration reduces the impact of scattering and absorption by non‐vascular tissues (e.g., bone, muscle), as well as ambient light interference.

## 3 Conclusion

This study introduces a heterogeneous alloy approach to design efficient NIR transparent electrodes by doping Bi into Mg. The resulting alloy forms a micro‐mesh‐like structure that confines the oscillation of free electrons, reduces reflectivity, and enhances transmittance. As a result, a semitransparent NIR electrode was successfully developed with a transmittance of nearly 40% in a wide wavelength range (400–1600 nm) and a sheet resistance of 29.3 Ω ◻^−1^. When combined with a capping layer in OLEDs, the transmittance increases to ∼60% in the organic‐to‐air optical configuration, which suppresses surface plasmon polariton modes and enhances radiative coupling into air modes. This enables the OCE of the NIR OLED to reach 42.3% and the PLQY to increase to 70%. With these advances, at 704 nm, flexible NIR OLEDs achieve a record EQE of 24.3% at an emitting area of 10 mm^2^ and 19.7% at an emitting area of 900 mm^2^. Finally, the practical application of the flexible Mg:Bi‐based NIR OLEDs was demonstrated in a wearable photoplethysmography sensor, capable of reliably detecting pulse signals.

## 4 Experimental Section

### 4.1 Materials

Di‐[4‐(N,N‐di‐p‐tolyl‐amino)‐phenyl]cyclohexane, 4,4′,4′′‐tri(N‐carbazolyl)‐triphenylamine, 4,4'‐bis(carbazol‐9‐yl)biphenyl, 1,3,5‐tri[(3‐pyridyl)‐phen‐3‐yl] benzene, molybdenum oxide, and lithium fluoride of OLED materials were purchased from Lumtech Corp. All materials were used as received without further purification.

### 4.2 Device Fabrication

Various OLED structures were fabricated on different substrates, and the specific parameters are explained in the main body. Glass substrates were cleaned with Decon 90 and ultrasonication in ultrapure water for 10 min. After that, the substrates were treated with air plasma for 5 min after being dried at 120°C for 10 min. Finally, anode, functional layers, and cathode materials were sequentially deposited on the substrates by a vacuum thermal evaporation process in a vacuum chamber (∼6 × 10^−4^ Pa). The patterns were defined through different metal masks. The deposition rates of the metal electrodes, inorganic materials, and organic materials are 1–2 Å s^−1^, 0.1–0.2 Å s^−1^, and 0.2–0.8 Å s^−1^, respectively.

### 4.3 Device Measurements and Film Characterizations

The refractive index (n) and extinction coefficient (k) used in the calculations were obtained via spectroscopic ellipsometry (M‐2000UI, J.A. Woollam, USA) using WVASE software, as shown in Figure S17a,b. To validate these optical constants, the transmittance of the films was simulated using TFCalc 3.5.6 (Software Spectra, Inc.) and the results were consistent with experimental data, as presented in Figure S17c,d. The optical transmittance and reflectivity spectra were acquired using the spectroscopic ellipsometer, while the sheet resistance measurements were performed with an ST‐21H four‐point probe system (4 Probes Tech, China). The Hall effect data were measured via Lake Shore Cryotronics Accent HL5500 Hall System. TEM measurements were acquired via Shiyanjia Lab (www.shiyanjia.com). The film samples were prepared by direct thermal evaporation onto a copper grid coated with an ultrathin carbon support film. Observations were carried out using an FEI Talos F200X (Thermo Fisher Scientific, USA) TEM operated at an accelerating voltage of 120 kV in bright‐field mode. For temperature‐dependent resistance characterization, precise temperature condition was achieved using the wide‐range temperature control sample holder (PR‐SH‐WTC1, customized, PURI Materials, China), which varied the sample temperature over a range from −30°C to 120°C. The resistance was determined by applying a 2 V DC bias using a Keithley 2400 source meter and measuring the current according to Ohm's law. The EL performance of the OLEDs was measured immediately after fabrication, without encapsulation, under ambient conditions using a gonio‐photometric measurement system equipped with an MCPD‐9800 array spectrometer (GP500, Otsuka Electronics Co., Osaka, Japan) and a Keithley 2400 source meter (Keithley Instruments, USA). The system was pre‐calibrated with a Deuterium‐Halogen Calibration Light Source (DH‐3, Ocean Optics). Reported values are averages from 3 devices (σ <5%). Figure S18 displays the noise characteristics of the detector used in the testing system. The performance parameters were calculated by the measurement software using the following equations:

$$\Phi_{v}=K_{m}\int_{350}^{1050}V\left(\lambda \right)\Phi_{e\lambda }d\lambda ,$$

$$I_{v}=\frac{\partial \Phi_{v}}{\partial \Omega }\;,$$

$$CE=\frac{I_{v}}{I}\;,$$

$$EQE=\frac{\pi q}{hc\cdot I}\;\int_{350}^{1050}\lambda \Phi_{e\lambda }d\lambda ,$$

where, *Φ_v_* (lm) denotes luminous flux, I_v_ (cd) is luminous intensity, CE (cd A^−1^) is current efficiency, K_m_ is the maximum spectral luminous efficiency, V(λ) is the luminosity function, λ is the wavelength, Ω is the solid angle, I is the current, V is the bias voltage, q is the elementary charge, h is the Planck's constant, c is the speed of light. The EQE was determined through angle‐resolved measurements, incorporating spectral and angular intensity corrections to account for non‐Lambertian emission characteristics. The photocurrent and dark current of the OPD were measured under voltage bias using a Keithley 2902 Source Measure Unit with current recorded via TestPoint software. AFM measurements of metal films were analyzed using tapping‐mode atomic force microscopy (AFM, Dimension Icon, Bruker). Optical simulations of OLEDs were conducted using custom‐made software OptiXLED. The simulation methodology is grounded in classical electromagnetic theory and dipole models, particularly incorporating the far‐field dipole radiation power formalism for OCE calculations [42]. The output waveforms of the PPG sensor were measured using a self‐designed signal conditioning circuit consisting of a transimpedance amplifier, a bandpass filter, an amplifier, and a low‐pass filter, which together convert, filter, and amplify the signal to extract clean physiological pulse waveforms [43].

## Conflicts of Interest

The authors declare no conflicts of interest.

## Supporting information




**Supporting File**: adma72640‐sup‐0001‐SuppMat.docx.

## Data Availability

The data that support the findings of this study are available from the corresponding author upon reasonable request.
