# Supplementary material for: High‐Performance Flexible Near‐Infrared Organic Light‐Emitting Diodes with a Heterogeneous Alloy Semitransparent Electrode
Source: Adv Mater. 2026 Feb 26;38(17):e18212. doi: 10.1002/adma.202518212 (PMC13003902; doi:10.1002/adma.202518212)
Supplement: Supplementary file 1 — Supporting File: adma72640‐sup‐0001‐SuppMat.docx. [file ADMA-38-e18212-s001.docx]

Supporting Information

**High-Performance Flexible Near-Infrared Organic Light-Emitting Diodes with Heterogeneous Alloy Semitransparent Electrode**

Mengxin Xu^1^, Meina Han^1^, Minying Xue^2^, Shihao Liu^1^*, Yi Li^1^, Gaoqiang Deng^1^, Letian Zhang^1^, Yuantao Zhang^1^, Gang Cheng^2,3^*, Wenfa Xie^1^*, and Chi-Ming Che^2,3^*

M. X. Xu, M. N. Han, S. H. Liu, L. T. Zhang, Y. Li, G. Q. Deng, Y. T. Zhang, W. F. Xie

^1^State Key Laboratory of Integrated Optoelectronics, JLU Region, College of Electronic Science and Engineering, Jilin University, Changchun 130012, China.

*E-mail: liushihao@jlu.edu.cn; xiewf@jlu.edu.cn

M. Y. Xue, G. Cheng, C. M. Che

^2^State Key Laboratory of Synthetic Chemistry, HKU-CAS Joint Laboratory on New Materials, Department of Chemistry, The University of Hong Kong, Pokfulam Road, Hong Kong SAR 999077, China.

*E-mail: ggcheng@hku.hk; cmche@hku.hk

G. Cheng, C. M. Che

^3^Hong Kong Quantum AI Lab Limited, Units 909-915, Building 17W, 17 Science Park West Avenue, Hong Kong Science Park, Pak Shek Kok, Hong Kong SAR 999077, China.

**Supplementary Figure:**

Figure S1. Transmittance spectra of Mg:Ag and Mg:Bi alloy films.

Figure S2. Hall mobility in Mg:Bi alloys.

Figure S3. Temperature-dependent resistance of pure Mg and Mg:40 wt% Bi alloy thin films.

Figure S4. XRD patterns of Mg:Bi alloys.

Figure S5. SAED patterns of Mg:Bi alloy films with 20 wt% and 80 wt% Bi.

Figure S6. Normalized EL spectra of D-Ag and D-MB at various viewing angles.

Figure S7. Schematic representation of the CL-Ag structure.

Figure S8. Transmittance spectra of Mg:Bi (20 nm) with and without TAPC (60 nm) capping layer in an organic-to-air optical configuration.

Figure S9. Normalized EL spectra of CL-60 at viewing angles of 0°, 40°, and 80°.

Figure S10. EL performance of CL-60 and CL-Ag.

Figure S11. Real part of the permittivity for semitransparent Ag and Mg:Bi alloy films.

Figure S12. Transient PL decay characteristics of non-cavity (C-Ag, C-MB) and microcavity (D-Ag, D-MB) devices.

Figure S13. EL performance of NIR OLEDs with Mg:Bi vs. Ag electrodes using the APDC-DTPA emitter (>750 nm).

Figure S14. EL performance of NIR OLEDs with Mg:Bi vs. Ag electrodes using the Pt 2-46 emitter (>850 nm).

Figure S15. Atomic force microscopy surface morphology of bent PET films.

Figure S16. Device structure and light-dark current density characteristics of the used organic photodetector.

Figure S17. Optical properties of Mg, Bi, Mg:Bi alloy, and Ag films.

Figure S18. Detector noise characteristics of the testing system.


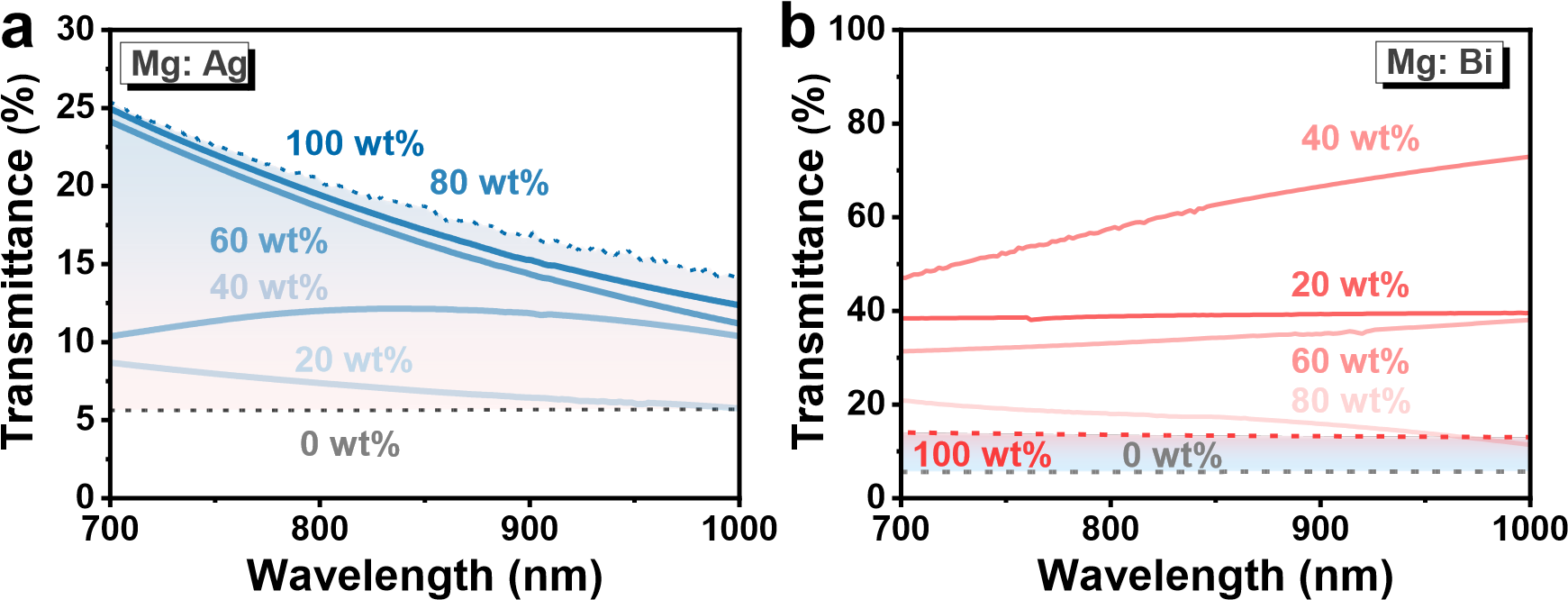


**Figure S1.** Transmittance spectra of Mg:Ag and Mg:Bi alloy films. Transmittance spectra of 20 nm films of (a) Mg:Ag alloy as a function of Ag ratio and (b) Mg:Bi alloy as a function of Bi ratio.

**Figure S2**. Mobility in Mg:Bi alloys with Bi ratios of 20 wt%, 40 wt%, 60 wt%, and 80 wt%, determined via Hall effect measurements.

**Figure S3.** Temperature-dependent resistance of (a) pure Mg and (b) Mg:40 wt% Bi alloy thin films. To ensure continuous film formation for the nominally pure Mg electrode, a 0.5-nm-thick Bi wetting layer was pre-deposited on the substrate prior to Mg evaporation.

**Figure S4.** X-ray diffraction (XRD) patterns of Mg:Bi alloy films with varying Bi concentrations. The evolution from Mg- to Bi-dominated crystal structures is shown, with peaks indexed to hexagonal Mg (red, PDF# 00-035-0821) and rhombohedral Bi (blue, PDF# 00-044-1246). Minor peaks from Bi₂O₂CO₃ are marked with grey asterisks.

**Figure S5.** SAED patterns of Mg:Bi alloy films with 20 wt% and 80 wt% Bi.

**Figure S6.** Normalized EL spectra of D-Ag and D-MB at various viewing angles. Normalized EL spectra of (a) D-Ag and (b) D-MB at viewing angles of 0°, 20°, 40°, 60°, and 80°.

**Figure S7.** Schematic representation of the CL-Ag structure.

**Figure S8.** Transmittance spectra of a 20 nm Mg:Bi film with and without TAPC (60 nm) capping layer in an organic-to-air optical configuration. The black solid line shows the measured transmittance of the 20 nm Mg:Bi film alone (air/Mg:Bi/air structure). The red solid line shows the measured transmittance of the stack with a TAPC capping layer (air/Mg:Bi/TAPC/air structure). The dashed line represents the simulated transmittance for the organic-medium/Mg:Bi/TAPC/air stack, modeling the actual device conditions where the incident medium is an organic layer. Since transmittance measurements are conducted with air as the incident medium, they may not fully represent the internal optical environment of the OLED, where the incident medium is an organic layer with a refractive index close to that of TAPC. Simulations using the organic-incident-medium model indicate that the effective transmittance under operational conditions can reach ~60%. The enhanced transmittance with the TAPC capping layer is attributed to refractive index matching, reduced reflection losses, and constructive interference within the multilayer structure. According to the Fresnel equations, lower refractive index contrast at interfaces minimizes reflection and enables a more symmetric optical field distribution, improving light coupling and transmission efficiency.

**Figure S9.** Normalized EL spectra of CL-60 at viewing angles of 0°, 40°, and 80°.

**Figure S10**. EL performance of CL-60 and CL-Ag. (a) J-V-R characteristics, (b) EQE-J characteristics, (c) EL spectra, and (d) angular emission profiles.

**Figure S11.** Real part of the permittivity for semitransparent Ag and Mg:Bi alloy films. Owing to its large negative permittivity, Ag supports strongly confined surface plasmon polariton (SPP) modes with a high wavevector *k*_SPP_, as given by $k_{SPP}=k_{0}\sqrt{\frac{\varepsilon_{m}\varepsilon_{d}}{\varepsilon_{m}+\varepsilon_{d}}}$, where *ε*_m_ and *ε*_d_ are the complex permittivity of the metal and the adjacent dielectric, respectively, and *k*_0_ is the vacuum wavevector. In contrast, the Mg:Bi alloy exhibits a smaller negative permittivity, resulting in more weakly confined SPP modes with smaller *k*_SPP_, which can be more efficiently outcoupled into free space, especially when assisted by a capping layer.

**Figure S12.** Transient PL decay characteristics of devices with different electrode structures. Transient PL decay characteristics of (a) C-Ag and D-Ag; (b) C-MB and D-MB. The structure of C-Ag: MoO_3_ (3 nm)/TAPC (80 nm)/TCTA (10 nm)/ CBP: Pt-4C (8 wt%, 20 nm)/TmPyPB (60 nm)/LiF (1 nm)/Ag (20 nm); C-MB: MoO_3_ (3 nm)/TAPC (80 nm)/TCTA (10 nm)/ CBP: 8 wt% Pt-4C (20 nm)/TmPyPB (60 nm)/LiF (1 nm)/Mg: Bi (20wt%, 20 nm).

**Figure S13**. EL performance of NIR OLEDs with Mg:Bi vs. Ag electrodes using the APDC-DTPA emitter (>750 nm). (a) Device structure, (b) J-V-R characteristics, (c) EQE-J characteristics, (d) EL spectra emission profiles of the TADF NIR OLEDs. The device structure is Al (100 nm)/MoO_3_ (3 nm)/N,N-bis(1-naphthyl)-N,N′-diphenyl-1,1′-biphenyl-4,4′-diamine (NPB, 90 nm)/TCTA (10 nm)/APDC-DTPA (20 nm)/1,3,5-tris(1-phenyl-1H-benzimidazol-2-yl)benzene (TPBi, 70 nm) /LiF (1 nm)/cathode (20 nm)/NPB (60 nm).

**Figure S14**. EL performance of NIR OLEDs with Mg:Bi vs. Ag electrodes using the Pt 2-46 emitter (>850 nm). (a) Device structure, (b) J-V-R characteristics, (c) EQE-J characteristics, (d) EL spectra emission profiles of the Pt 2-46-based NIR OLEDs. The device structure is Al (100 nm)/FSFA: NDP-9 (3 wt%, 10 nm)/FSFA (130 nm)/NBP-BC (5 nm)/RH: Pt2-46 (12 wt%, 40 nm)/1-[4-(10-([1,1-biphenyl]-4-yl) anthracen-9-yl) phenyl]-2-ethyl-1H-benzo[d] imidazole (ANT-Biz, 5 nm)/ANT-Biz: 8-Quinolinolato lithium (Liq) (3 wt%, 30 nm)/LiF (1 nm)/cathode (20 nm)/FSFA (60 nm).


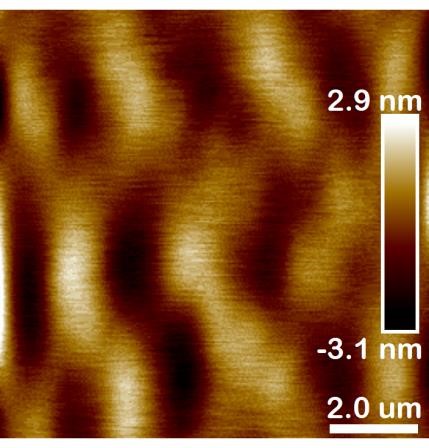

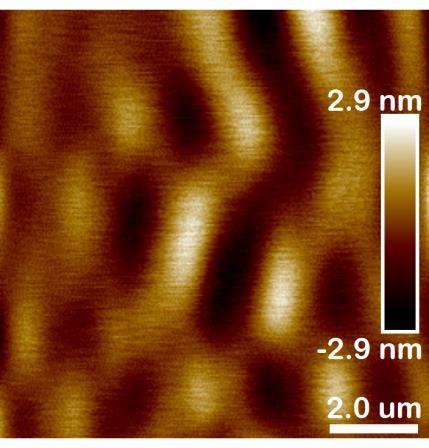


**0**

**circles**

**1000**

**circles**

**Figure S15.** Atomic force microscopy surface morphology of bent PET films.

|  |  |
| --- | --- |

**Figure S16.** Device structure and light-dark current density characteristics of the used organic photodetector. (a) Device architecture of the OPD. (b) Light and dark current density as a function of bias voltage. The OPD structure is Al (100 nm)/MoO_3_ (5 nm)/copper(II) phthalocyanine (CuPc, 20 nm)/ CuPc:C_60_ (50 nm, 50 wt%)/C_60_ (20 nm)/2,9-Dimethyl-4,7-diphenyl-1,10-phenanthroline (BCP, 10 nm)/4,7-Diphenyl-1,10-phenanthroline, (Bphen, 30 nm)/Mg:Bi (20 nm, 20 wt%). Photocurrent measurements were performed using a calibrated NIR LED source with a radiant power of 10 mW, matching the peak emission wavelength of the NIR OLED.

**Figure S17.** Optical properties of Mg, Bi, Mg:Bi alloy, and Ag films. (a) Refractive index (*n*) and extinction coefficient (*k*) of Mg:Bi alloys with varying Bi concentrations as a function of wavelength. (b) Wavelength-dependent *n* and *k* values of Ag. (c) Comparison between fitted and experimental transmittance spectra of Mg:Bi alloys. (d) Comparison between fitted and experimental transmittance spectra of Ag.

**Figure S18**. Detector noise characteristics of the testing system. (a) Raw detector noise characteristics. (b) D-MB spectrum comparison with noise signal.
